# Objective cough frequency in Idiopathic Pulmonary Fibrosis

**DOI:** 10.1186/1745-9974-6-4

**Published:** 2010-06-21

**Authors:** Angela L Key, Kimberley Holt, Andrew Hamilton, Jaclyn A Smith, John E Earis

**Affiliations:** 1 Respiratory Department, Aintree University Hospitals NHS Foundation Trust, University Hospital Aintree, Longmoor Lane Liverpool, L9 7AL, UK; 2 Respiratory Research Group, School of Translational Medicine, University of Manchester, 2nd Floor, Education and Research Centre, Wythenshawe Hospital, Southmoor Road, Manchester M23 9LT, UK; 3 School of Environment and Life sciences, University of Salford, Salford, Greater Manchester, M5 4WT UK

## Abstract

**Background:**

Cough is a common presenting symptom in patients with Idiopathic Pulmonary Fibrosis (IPF). This study measured cough rates in IPF patients and investigated the association between cough and measures of health related quality of life and subjective cough assessments. In addition, IPF cough rates were related to measures of physiological disease severity and compared to cough rates in health and other respiratory conditions.

**Methods:**

Nineteen IPF patients, mean age 70.8 years ± 8.6, five female (26.3%) were studied. Subjects performed full pulmonary function testing, 24 hour ambulatory cough recordings, completed a cough related quality of life questionnaire (Leicester Cough Questionnaire) and subjectively scored cough severity with a visual analogue scale. Ambulatory cough recordings were manually counted and reported as number of coughs per hour.

**Results:**

The 24hr cough rates were high (median 9.4, range 1.5-39.4), with day time rates much higher than night time (median 14.6, range 1.9-56.6 compared to 1.9, range 0-19.2, p = 0.003). Strong correlations were found between objective cough frequency and both the VAS (day r = 0.80, p < 0.001, night r = 0.71, p = 0.001) and LCQ (r = -0.80, p < 0.001), but not with measures of pulmonary function. Cough rates in IPF were higher than healthy subjects (p < 0.001) and asthma patients (p < 0.001) but similar to patients with chronic cough (p = 0.33).

**Conclusions:**

This study confirms objectively that cough is a major, very distressing and disabling symptom in IPF patients. The strong correlations between objective cough counts and cough related quality of life measures suggest that in IPF patient's, perception of cough frequency is very accurate.

## Background

Idiopathic Pulmonary Fibrosis (IPF) is a progressive fibrotic disease of unknown aetiology with an estimated incidence of 6-20 cases per 100,000 of the population. Clinical features include dry cough, breathlessness, restrictive spirometry, end-inspiratory crackles, reduced oxygenation and finger clubbing. High resolution computer tomography (HRCT) shows a distinctive pattern of sub pleural shadowing and later in the disease honeycomb fibrosis [1]. Cough in IPF, is both a presenting and a complicating clinical feature [2].

Swigris *et al *conducted in-depth interviews with IPF patients to determine how the disease affects their lives [3]. They described cough as being dry, nonproductive and hacking with significant physical and social impacts on their lives. Moreover there is often a constant urge to cough which was unrelieved by coughing [3]. Other reports confirmed these observations and suggest that cough affects 73-86% of cases [4,5].

To date there are no studies objectively quantifying cough in IPF patients. The aims of this study were to measure cough rates in subjects with IPF and investigate the relationships between objective cough rates, subjective cough assessments and cough related quality of life. Any association between cough rates and measures of disease severity were also explored. Finally cough rates in IPF were compared to previously published data, collected using identical methodology in healthy controls, asthma and isolated chronic cough [6,7].

## Methods

### Subjects

Nineteen patients were recruited from two specialist Interstitial Lung Disease (ILD) clinics at University Hospital Aintree (UHA) and University Hospital South Manchester (UHSM). All patients met ATS/ERS criteria for the diagnosis of IPF i.e. demonstrated typical clinical, spirometric and radiological changes consistent with IPF [8]. Subject selection was not based on the presence of coughing. Patients taking ACE inhibitors, opiates or other antitussive medications, and those that had suffered from a respiratory tract infection within 8 weeks were excluded. A sample size of 20 subjects would have approximately 80% power to detect correlation coefficients of 0.55 and above [9]. Ethical approval was obtained from the relevant Local Ethics Research Committees prior to the study commencing (UHA - St. Helens and Knowsley Local Research Ethics Committee (reference: 05/Q1508/43) and UHSM-South Manchester Research Ethics Committee (reference: 06/Q1403/128)) and all patients provided informed written consent. Research was carried out in compliance with the Helsinki Declaration.

### Assessment of Pulmonary Function

All subjects underwent full pulmonary function testing including gas transfer tests and body plethysmography (UHA - Zan Messgerate Body plethysmograph 530, UHSM - Vmax, Sensor Medics).

### Objective Measures of Cough

Patients underwent 24 hour ambulatory cough sound recording as previously described [6,7,9-12] (Vitalojak, Vitalograph Ltd, Buckingham, UK). To establish repeatability, 24 hour cough counts were measured on two occasions in 11 patients. Sound files were recorded onto a 4GB data card and transferred to a personal computer for analysis. Manual cough counting was performed by trained staff using a wave-editing package with an audiovisual display (CoolEdit 2000, Syntrillium Software corp., AZ, USA). The numbers of explosive phases of the cough sounds were counted and hourly rates calculated. The explosive phase was a characteristic irregular and noise-like waveform and was readily differentiated from the regular (periodic) waveform of voiced sounds [13]. Repeatability of manual cough counting was established by a second investigator who re-counted five 30 minute segments of each recording.

### Subjective Measures of Cough

#### Cough VAS

All patients were asked to mark cough severity on a linear 100 mm visual analogue scale for the day and for the night time. The extremes of the scale were marked from 'no cough' to 'worst cough'.

#### Leicester Cough Questionnaire

The Leicester Cough Questionnaire (LCQ) is a validated, reproducible, 21 item self completed questionnaire. These 21 questions are sub-divided in to three domains; social, psychological and physical. The total calculated score ranges from 3-21; a higher score indicates a better quality of life [14].

### Statistical Analysis

Analysis was carried out using SPSS Version 15.0 (SPSS inc., IL, USA) and Prism 4 (Graphpad Software Inc., CA, USA). Where appropriate, parametric data are reported (mean ± standard deviation). Repeatability of cough counting in IPF and the reproducibility of cough counts were assessed by the method described by Bland and Altman [15]. Cough rate data was not normally distributed, therefore for correlations non-parametric testing was applied (Spearman correlation coefficient). In order to compare cough rates from previously published data in healthy controls, asthma and chronic cough, rates were logarithmically transformed (base 10) and a one-way ANOVA performed.

## Results

### Subjects

Nineteen IPF patients, diagnosed between 2001 and 2007, were studied (Table 1). Seven patients had never smoked; the remaining 11 were ex smokers (median pack years 20 range 4.2-52). Thirteen patients were taking oral steroids at the time of study (68%). Pulmonary function demonstrated a typical restrictive pattern of ventilation and reduced DLco. One patient was unable to obtain a TLC using plethysmography.

**Table 1:** Patient demographics

Characteristic	Value
Age (years)	70.8 (± 8.6)

Gender (% female)	5 (26.3%)

BMI (kg/m^2^)	28.5 (23.5-36.21)

IPF Duration (years)	3 (1-6)

FEV_1 _(% predicted)	78.3% (± 20.9)

FVC (% predicted)	78.5% (± 24.4)

FEV_1 _/FVC (%)	75.8% (± 4.3)

TLC (% predicted)	68.1% (± 20.19)

DLco (% predicted)	43.2% (± 16.06)

Kco (% predicted)	77.2% (± 16.65)

Steroid Use	68%

Of these 19 patients, two of were found to have HRCT evidence of upper lobe emphysema in addition to typical changes of IPF. However, only one showed any spirometric changes in keeping with airflow obstruction.

### Objective measures of cough

**Figure 1:**
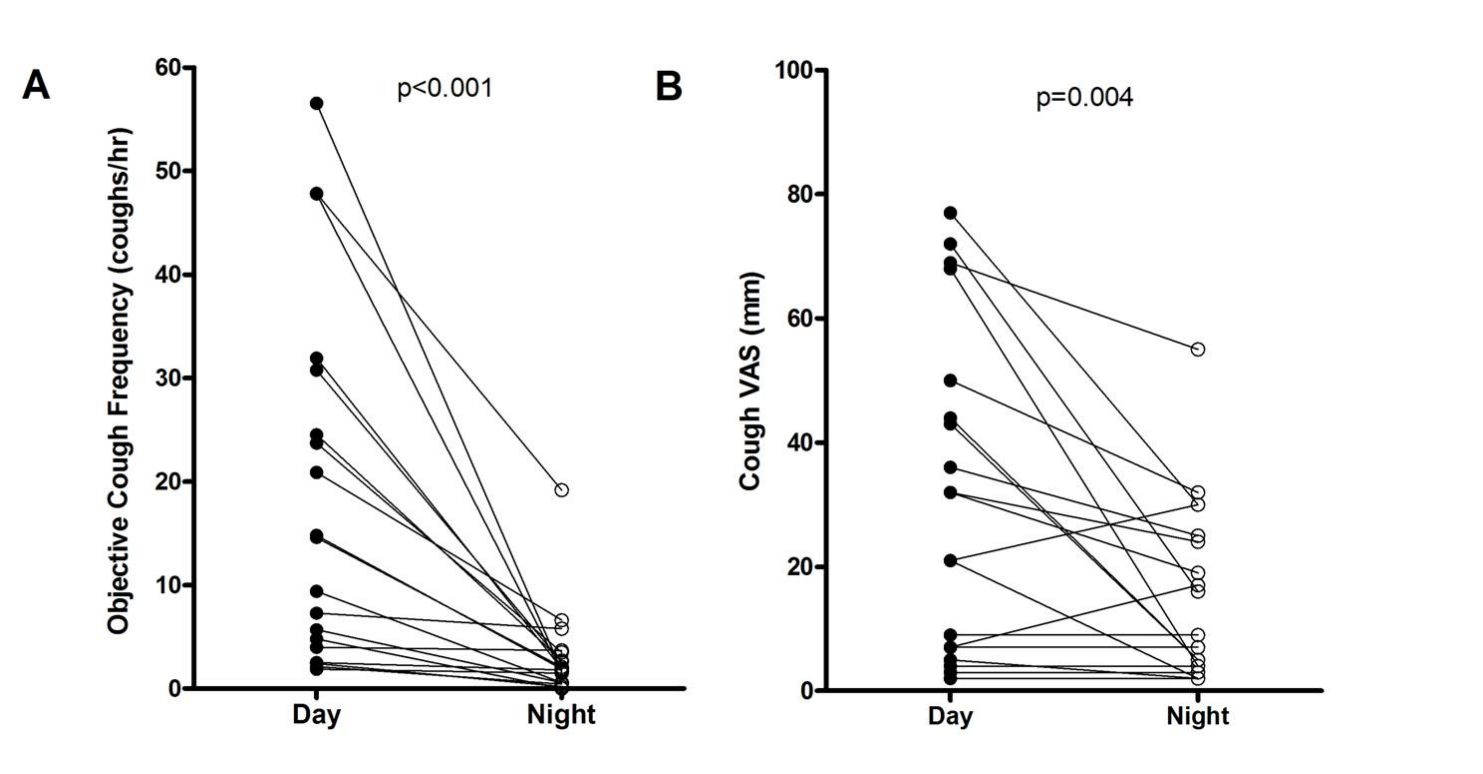
**Objective cough frequency (A) and cough VAS scores (B) for day and night in patients with IPF**.

The overall 24 hour cough rates were high (median 24 hour cough rate 9.4 per hour, range 1.5-39.4), with the day time rates much higher than night time (median 14.6, range 1.9-56.6 compared to 1.9, range 0-19.2, p = 0.003), see figure 1A. Day cough rates moderately correlated with the night time cough rate (r = 0.47, p = 0.04) but neither gender nor age (p = 0.69 and p = 0.33 respectively) were related to cough rates. One analysis in this study was to stratify the data into those who were taking steroids and those who were not and there were no significant differences between the groups (day p = 0.97, night p = 0.71). Patients smoking pack year history also showed no relationship with cough rates (day p = 0.418, night p = 0.533). The first 11 patients were recorded twice to check the reproducibility of the cough measurements (median 11 days apart (IQR 9.25- 40.5)). Bland Altman plots demonstrated cough rates were highly reproducible over time (mean difference in 24hr cough rate 0.3 ± 5.2). In addition, there was very good agreement between the two observers, with a mean difference of only 0.9 (± 1.7) coughs per hour,

### Subjective measures of cough

#### Cough VAS

The VAS was significantly higher for day (median 32 mm, range 2-77 mm) than for night (median 9 mm, range 2-55 mm, p < 0.001), Figure 1B.

For the group of patients recorded on two occasions the VAS score also demonstrated very stable results over time for both the day (mean difference 5.45 mm, ± 4.71 mm) and night (mean difference 4.82 mm, ± 9.67 mm).

#### Cough related quality of life

The median total LCQ score was 15.4 (6.95-20.88); median domain scores were physical 5.13 (2.38-6.63), psychological 5.29 (1.57-7) and social 5.75 (2.25-7). Again the 11 patients recorded on two occasions showed excellent agreement over time (mean difference, physical 0.22, psychological 0.30, social 0.05 and total 0.56). Age and gender did not significantly influence the LCQ scores (total LCQ p = 0.137 and p = 0.824 respectively).

### Relationships between Objective Cough Frequency, Cough VAS and Quality of Life

**Figure 2:**
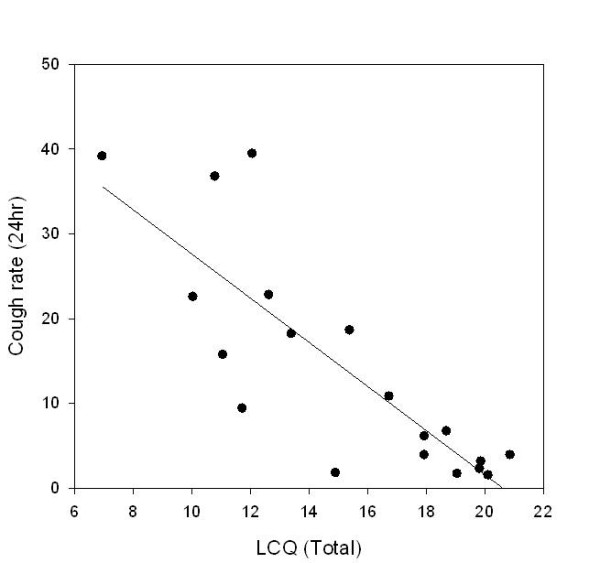
**Objective cough frequency (24 hour cough rate) and the total score from the LCQ**.

Strong correlations were found between objective cough frequency and both the cough VAS and cough related quality of life, see table 2 and Figure 2. Correlations tended to be stronger during the day than overnight and were also present for all domains of the LCQ.

**Table 2:** Relationships between objective and subjective cough measures

		Total Cough Rate	Day Cough Rate	Night Cough Rate
LCQ	Total	r = -0.80 p = < 0.001	r = -0.77 p = < 0.001	r = -0.50 p = 0.028
	
	Physical	r = -0.76 p = < 0.001	r = -0.72 p = 0.001	r = -0.46 p = 0.048
	
	Psychological	r = -0.76 p = < 0.001	r = -0.72 p = 0.001	r = -0.46 p = 0.048
	
	Social	r = -0.74 p = < 0.001	r = -0.71 p = 0.001	r = -0.55 p = 0.016

VAS	Day		r = 0.80 p = < 0.001	- -
	
	Night		- -	r = 0.71 p = 0.001

Spearman's Correlation Coefficients

### Relationships between Objective Measures of Cough and Measures of Disease Severity

In contrast, there were no significant correlations between cough rates and FEV^1^, FVC, DLco or Kco. A single positive correlation exists between TLC (percent predicted) and the total cough rate (r = -0.470, p = 0.049) i.e. suggesting higher TLC was associated with a lower frequency of cough.

### Comparisons with Objective Cough Frequency in other conditions

**Figure 3:**
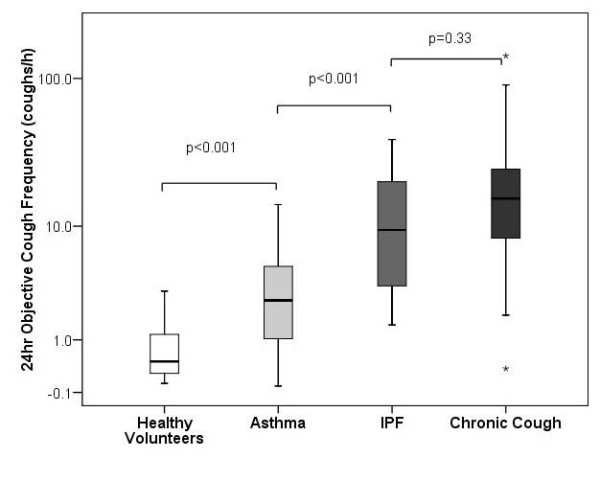
**Comparison of objective cough frequency in patients with IPF to previously published data in healthy volunteers,**[6]**asthma**[6]**and patients presenting with chronic cough**.

One-way ANOVA found significant differences between log10 objective cough frequencies in healthy volunteers (n = 18) [6], asthma (n = 56) [6], chronic cough (n = 86) [7] and IPF (n = 19), p < 0.001, see figure 3. Post-hoc analysis (Scheffe correction for multiple comparisons) suggested that cough rates in IPF were higher than healthy volunteers (p < 0.001) and asthma patients (p < 0.001) and similar to patients presenting to a specialist clinic with chronic cough (p = 0.33).

## Discussion

This study is the first to quantify cough in IPF patients and examine the relationships between objective cough rates and subjective measures. The IPF patients had frequent coughing, especially during the day, with relatively little nocturnal cough. Cough rates were wide ranging, did not appear to be influenced by the patient's age or gender and were highly reproducible between investigators and over a short period of time. A comparison between objective cough rates in IPF with other conditions associated with cough, showed that mean cough rates in this group of IPF patients were higher than in asthma but comparable to rates found in patients with an isolated chronic cough presenting to a specialist clinic.

In this study two subjective assessments of cough were employed, the LCQ and a Visual Analogue Scale. The LCQ is a fully validated questionnaire that provides an estimation of the physical, psychological and social impact of cough as well as providing a measure of the overall impact of coughing. In contrast the VAS is a simple measure that estimates the global impact of cough during the day and night. There was a strong and statistically significant relationship with cough rates and both of these measures. The LCQ correlated with both day and to a lesser degree night time coughing for all the domains. In view of the small numbers of coughs this night-time association was not expected. However, it is known that IPF is associated with sleep fragmentation and thus it is likely that any episode of nocturnal coughing will wake these patients [16].

The VAS performed similarly to the LCQ. The daytime VAS score (mean 33.40 mm, ± 25.33) is comparable to that from previous data in IPF (mean IPF 30 mm ± 19 and 40 mm ± 25 mm [17,18],). These strong correlations between objective cough counts and cough related quality of life measures suggest that in IPF patients perception of cough frequency is very accurate. These relationships appear to be stronger than those reported by patients presenting specifically with the symptom of chronic cough. Perhaps heightened awareness of coughing occurs in the context of breathlessness, or because coughing from restricted lung volumes requires greater effort. It is interesting that Doherty *et al *did not find an association between cough VAS and cough reflex sensitivity measured by capsaicin challenge [16], suggesting that the cough reflex sensitivity in IPF is not a very sensitive predictor of actual cough rates.

Exploring the association between cough frequency and measures of disease severity using pulmonary function showed no correlation between more advanced disease (e.g. lower TLC and DLco) and cough rates. In fact the only weak positive correlation was with total cough rates and higher TLC%. This is likely to be a chance result as the study was not designed nor powered to look for this association. However, the fact that there is no strong correlation between pulmonary function and cough rates suggests that cough is an established and troublesome symptom by the time patients present with this disease. A larger study is required to look at this association but these results suggest that cough may not be a good surrogate for disease progression.

The exact mechanisms underlying cough in IPF remain unclear, in particular why coughing should occur when the cough receptors are proximal whilst the main disease process is in the parenchyma. Several theories have been proposed to explain this observation and the similarity between the cough rates in IPF and chronic cough patients raises the possibility that there may be similar mechanisms at work in these two apparently disparate groups. For example, Irwin suggested that in a group of patients with known ILD referred to a specialist cough clinic, 50% had evidence of asthma, nasal or gastrooesophageal reflux disease (GORD);[18] GORD in particular is thought to be highly prevalent in IPF [19]. However, it is equally possible that IPF directly causes cough as inflammation is not limited to the parenchyma and airway inflammatory mediators known to provoke cough have been detected [20]. In keeping with this suggestion increased levels of albumin in the sputum of IPF patients have been reported which suggests disrupted airway epithelium [17]. Such damage is accompanied by neutrophilic inflammation, raised levels of nerve growth factor and brain derived neurotrophic factor [21]. A further possible cause of cough in these patients is airway distortion secondary to interstitial fibrosis which results in traction bronchiectasis.

## Conclusions

The correlations between subjective and objective measures of cough in this study were very strong, despite the relatively small sample size. For the first time this study confirms objectively that cough is a major, very distressing and disabling symptom in patients suffering from IPF. Currently treatment is not effective and a larger study, including longitudinal data, is needed to investigate the underlying mechanisms, trigger factors and treatment options for this group of very disabled patients.

## Competing interests

Dr Jacky Smith has no financial conflict of interest but is an inventor on a patent describing novel techniques for automated cough detection. This patent is owned by the University Hospital of South Manchester and is licensed to Vitalograph Limited.

## Authors' contributions

ALK has contributed to the design and conception of the study, data collection, analysis and interpretation and drafting/revising of the manuscript. KH and AH both contributed to data collection, analysis and interpretation. ALK, KH and AH were involved in drafting and revising the manuscript. JAS and JEE were both involved in the study design and conception. They were also involved in the analysis and interpretation of the data and had substantial input regarding the drafting and revision of the manuscript. All authors have read and approved the final version.
